# Insight into the Functionality of Microbial Exopolysaccharides by NMR Spectroscopy and Molecular Modeling

**DOI:** 10.3389/fmicb.2015.01374

**Published:** 2015-12-08

**Authors:** Flemming H. Larsen, Søren B. Engelsen

**Affiliations:** Spectroscopy and Chemometrics, Department of Food Science, University of CopenhagenFrederiksberg, Denmark

**Keywords:** gellan, diutan, bacterial exopolysaccharides, NMR spectroscopy, molecular modeling, molecular hydration

## Abstract

Microbial polysaccharides represent an important class of microbial polymers with diverse functions such as biofilm formation, thickening, and gelling properties as well as health-promoting properties. The broad range of exopolysaccharide (EPS) functionalities has sparked a renewed interest in this class of molecules. Chemical, enzymatic as well as genetic modifications by metabolic engineering can be used to create large numbers of analogous EPS variants with respect to EPS functionality. While this top–down approach is effective in finding new candidates for desired functionality, there seems to be a lack of the corresponding bottom–up approach. The molecular mechanisms of the desired functionalities can be established from Nuclear Magnetic Resonance (NMR) and molecular models and it is proposed that these models can be fed back into the biotechnology by using a quantitative structure–property approach. In this way it will be possible to tailor specific functionality within a given design space. This perspective will include two well-known commercial microbial EPS examples namely gellan and diutan and show how even a limited use of multiphase NMR and molecular modeling can increase the insight into their different properties, which are based on only minor structural differences.

## Introduction

Exopolysaccharides (EPSs) is a very diverse group of polysaccharides produced by a broad range of microbes, plants, and vertebrates. In natural environments EPS may constitute the main part of, e.g., biofilms, consisting of DNA, proteins, and several EPSs (Flemming and Wingender, 2010), but under controlled settings bacterial strains can produce highly specific types of polysaccharides (Coleman et al., 2008; Schmid et al., 2014). According to a recent review (Freitas et al., 2011), applications of EPS are increasing within the food, pharmaceutical, and petroleum industries. In the food industry several EPS are used as food ingredients, serving as emulsifiers, thickeners or gelling agents due to their ability to form hydrocolloids in aqueous solutions. Moreover, some EPS such as those produced by *Bifidobacteria* have probiotic characteristics (Salazar et al., 2008). EPS production may also have negative impact with their role in the formation of biofilms and biofouling, where the EPS provide a matrix on which bacteria can adhere and grow.

Modern biotechnology enables the use of genetically modified bacteria to produce large numbers of potentially different EPS samples. Obtaining a desired or new functionality can be achieved using high throughput functionality screening systems, but will normally not include molecular knowledge on how to correlate structure and function for analogous series of EPS with smaller and bigger differences. Accordingly, too little is known about the EPS and there is thus a need to develop an understanding of structure–function relationships, i.e., to relate EPS structure with rheological properties of their aqueous solutions, in order to exploit the use of EPS in new applications as well as during the production of fermented products. Detailed knowledge of the structures of EPS will furthermore help in determining the pathways by which the EPSs are synthesized *in vivo*.

**FIGURE 1:**
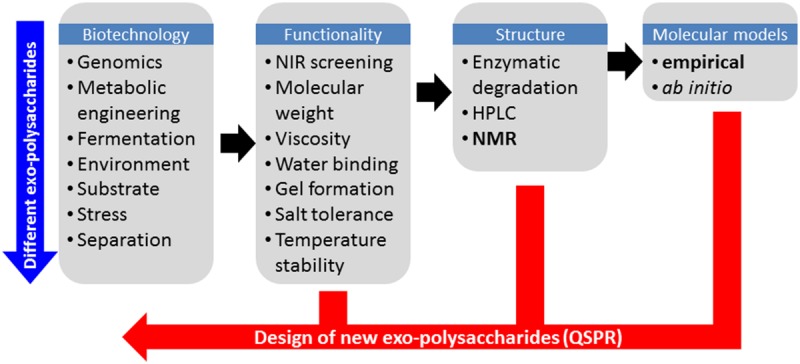
**Schematic overview of the work flow in tailoring exopolysaccharide functionality by first principles and by Quantitative Structure–Property Relationships (QSPRs)**.

One of the main challenges in systematic production of EPS with new functional characteristics is to find suitable rapid analytical solutions to probe the molecular structure. In this context, EPS present two major challenges: firstly the extreme flexibility of carbohydrates often makes them difficult to characterize experimentally, and secondly the high molecular weight (MDa range), high viscosity and low water solubility associated with EPS are major obstacles for the majority of analytical methods and in particular liquid-state ^1^H Nuclear Magnetic Resonance (NMR) which in many other respects would be the ideal method to study carbohydrate conformation in solution. Other analytical techniques can provide useful information about the secondary structure of EPS in solution such as small angle x-ray scattering (Yuguchi et al., 1996; Dogsa et al., 2008) and multi-angle light scattering (Tuinier et al., 2001) from which parameters such as radius of gyration and hydrodynamic ratio can be inferred. However, these techniques also suffer from the requirement of careful analytical sample preparations (purification, dilution, etc.). In fact only vibrational spectroscopy based techniques such as for example near infrared (NIR) spectroscopy has been reported to provide information that is largely independent of the molecular weight and state of the polymer, but unfortunately with less molecular details than more advanced analytical methods. Nevertheless the NIR method has proven to be a very efficient screening tool in phenomics and for detecting differences in biopolymer structure (Khakimov et al., 2014), and is thus an ideal pre-screening method for investigating if an EPS has been produced, and if so, if it is different from previous analyzed forms. An overall strategy to screen and test new EPS for a desired functionality is outlined in **Figure 1**.

When a new EPS is detected detailed knowledge about the biopolymer structure is required and NMR spectroscopy is irreplaceable for two reasons. Firstly, it can provide detailed information about the composition, connectivity, and conformation of the carbohydrate biopolymer, and secondly it can do so for aqueous solutions (Duus et al., 2000). No other analytical technique is capable of this and for carbohydrates and particularly carbohydrate polymers, it is their detailed hydrations that determines their conformation and therefore their resulting functionality (Kirschner and Woods, 2001). Liquid-state ^1^H NMR is for example the “golden reference method” for determining alginate functionality. The alginate polysaccharide is a simple linear polymer with repeating units of 1,4-linked β-D-mannuronic acid (M) and α-L-guluronic acid (G) arranged in a blockwise pattern along the polymer chain as homopolymeric (MM or GG) or heteropolymeric (MG) regions. The alginate functionality depends on the average molecular weight and on the distribution of the M- and G-units and on cations present. A low M/G-ratio typically results in a brittle gel, while a high M/G-ratio results in a softer and more flexible gel (Salomonsen et al., 2009). In this respect the linear ABABA-type alginate polymer is not very different from the complex hetero-polysaccharides, made from assembled repeating units. The only downside of liquid-state NMR is the requirement of depolymerised EPS samples using a stepwise hydrolysis. Secondly liquid-state NMR cannot be used as a stand-alone technique to study the detailed hydration of the polysaccharide. In order to achieve this, a combination of molecular modeling and multiphase NMR is required.

The combination of NMR and molecular modeling has proven a powerful tool for detailed characterization of the polymer structure and hydration for plant polysaccharides such as starch and pectin (Braccini and Perez, 2001; Damager et al., 2010; Larsen et al., 2011). However, also when it comes to molecular modeling, carbohydrates are a special case because of their extreme flexibility and the differences in their electronic arrangements such as the anomeric, exo-anomeric, and gauche effects (Pérez et al., 1996). Because of these inherent complex carbohydrate properties a pragmatic approach is required for modeling of polysaccharide structures. For this reason, software tools for structural calculation and visualization approaches of polysaccharides have been developed (Engelsen et al., 1996, 2014). This type of software can be used to study the packing of complex branched homo-polysaccharides such as starch (Damager et al., 2010) and for studying the helical structure of complex linear bacterial EPSs based on heterologous repeat units (Robijn et al., 1996). Evidently, the elucidation of structural and functional properties of polysaccharides are intimately linked to their interactions with water and NMR techniques provide an effective tool for to investigate the hydration of complex solutes such as aqueous EPS solutions.

This perspective aims to demonstrate the utility of a combined multiphase NMR and molecular modeling approach for the characterization of two microbial sphingan EPSs: gellan and diutan gum. Gellan has been by far one of the most studied EPS and include X-ray as well as NMR analysis (Jansson et al., 1983; Grasdalen and Smidsrod, 1987; Chandrasekaran et al., 1988; Bosco et al., 2000), whereas only few studies included NMR analysis of diutan (Evans et al., 2000). Even though X-ray analysis provides very detailed information about crystalline samples, multiphase NMR enables structural characterization of molecules present in crystalline and amorphous solids as well as gels and solutions. Since applications of hydrocolloids are often related to their gel or semi-solid state the molecular characteristics in these states are very important. Therefore, the combination of multiphase NMR with molecular modeling represents a powerful tool for the characterization of EPS’s in their true state of application.

## Materials and Methods

### Samples

Samples were kindly provided by Jochen Schmid, Technische Universität, München, Germany, and used without further purification. The gellan gum was Kelcogel F and the diutan gum was Kelco-Crete DG. Both were manufactured by CP Kelco.

### NMR Analysis

Samples for liquid-state NMR spectroscopy were prepared by mixing 1.6–2.3 mg of powder with 1200 μL D_2_O (containing TSP-d4). Liquid-state NMR was performed on a Bruker Avance DRX 500 spectrometer operating at 500.13 MHz for ^1^H using a double-tuned BBI probe equipped for 5 mm (o.d.) sample tubes. All experiments were performed at 80°C using a single-pulse experiment (90°degree flip angle), a recycle delay of 5 s, an acquisition time of 1.64 s and a spectral width of 10 kHz. All spectra were referenced to TSP-d4 at 0.0 ppm.

Solid-state NMR experiments were performed on a Bruker Avance 400 spectrometer operating at 400.13 and 100.62 MHz for ^1^H and ^13^C, respectively; using a double-tuned solid-state NMR probe equipped with 4 mm (o.d.) rotors. Both ^13^C CP/MAS NMR spectra were recorded at room temperature using a spin-rate of 9 kHz, a contact time of 1 ms, a recycle delay of 8 s, 1024 scans and an acquisition time of 49.3 ms during which high power ^1^H decoupling was applied. The spectra were referenced to α-glycine (external sample) at 176.5 ppm.

### Molecular Modeling

Models of the two EPSs gellan and diutan were constructed using the molecular modeling software POLYS (Engelsen et al., 1996, 2014). This program constructs the polysaccharides from their primary structure by combining information about the constituting monosaccharides with information about the glycosidic linkage geometries. In molecular models of polysaccharides, the two torsional angles (ϕ, Ψ) describing the glycosidic linkage (three for 1-6- linkages) are the most important structural degrees of freedom and the conformational parameters that determine the overall geometry of the polysaccharide. The glycosidic linkage torsional angles ϕ and Ψ, which have the definition ϕ = O5-C1-O-C′, and Ψ = C1-O-C′_X_-C′_(X-1)_ for a (1-X) linkage. In this study the data for the glycosidic linkages was adapted from the fiber-diffraction data of Chandrasekaran et al. (1988). Using these data the structures of the two EPS gellan and diutan were constructed using a simple nomenclature and subsequently optimized to the nearest threefold helical geometry (Pérez et al., 1996).


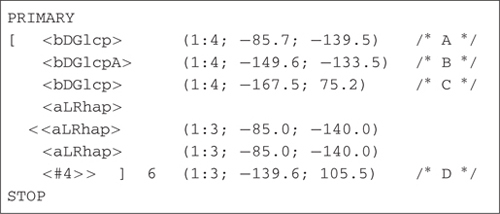


## Results and Discussion

**FIGURE 2:**
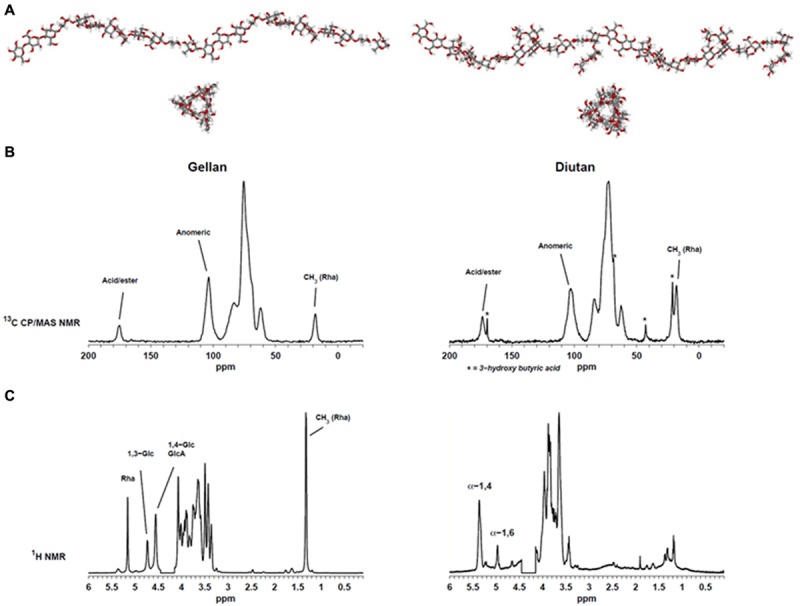
**(A)** Molecular modeling of gellan (left) and diutan (right). **(B)** Solid-state ^13^C CP/MAS spectra of gellan (left) and diutan (right). **(C)** ^1^H Liquid-state Nuclear Magnetic Resonance (NMR) spectra of gellan (left) and diutan (right). The partly suppressed water resonance (4.15–4.45 ppm) was removed from the ^1^H Liquid-state NMR spectra.

Determination of the 3D-structures of macromolecules can be very time consuming. Often it requires preparation of crystalline materials which may not always be possible for polysaccharides. Two different EPS were analyzed in this study. Gellan, which has a backbone of [→4) α-L-Rha (1→3) β-D-Glc (1→4) β-D-GlcA (1→4) β-D-Glc (1→], and diutan with a similar backbone except that the β-(1→4)-linked glucopyranose has a sidechain of a α-(1→4)-linked L-Rha disaccharide attached to C3. Gellan was characterized by X-ray diffraction (Chandrasekaran et al., 1988) and shown to be organized (in solid state) in two left-handed threefold helices. Construction of molecular models of the two polysaccharides gellan (heterologous linear repeat polysaccharide) and diutan (heterologous branched polymer) can efficiently generate visual models (**Figure 2A**) which can be used to explain NMR data and/or molecular functionality. It is for example obvious that the side chain in diutan do not extend the highly probable threefold structure of the gellan backbone and thus do not entangle in complex condensed phase which may indicate that the diutan molecules will still be able to align and create chain–chain interactions (Pérez et al., 1996). However, on the other hand the sidechain may suffice to prevent stronger junction zones (double helical interaction) and thus more likely to create thickening properties, but does not show the same strong gel-behavior like gellan.

**Figure 2** shows the ^1^H liquid-state and ^13^C CP/MAS NMR spectra of the two EPS. The solid-state NMR spectra (**Figure 2B**) display resonances from the methyl group in rhamnose at 18.1 ppm, resonances from CHOH and CH_2_OH carbons in the carbohydrate residues in the region 50–110 ppm and resonances from acid carbonyls in GlcA at 175.2 and 173.6 ppm, respectively. Moreover, a series of narrow resonances (21.2, 42.7, 68.4, 169.7 ppm) in the sample of diutan suggests the presence of 3-hydroxy butyric acid (Orgambide et al., 1991). The absence of an intense resonance at ∼21 ppm indicates that the β-1,3-linked glucose in the backbone was not O-acetylated and also no significant presence of O-glycerate was detected in the gellan sample.

The corresponding liquid-state NMR spectra (**Figure 2C**) provide a much higher spectral resolution compared to the solid-state NMR spectra, but only the mobile parts of the samples (i.e., no junction zones) are detected. With regard to the gellan spectrum it is similar to previously reported spectra and besides the resonance from methyl in rhamnose at 1.32 ppm, three resonances in the region 4.5–5.5 ppm could be assigned to anomeric hydrogens from rhamnose (5.16 ppm), glucose (4.73 (1→3); 4.55 (1→4) ppm) and galacturonic acid (4.55 ppm) (Bosco et al., 2000). Structurally, diutan is very similar to gellan and only differ by the addition of a rhamnose disaccharide side chain on the β-1,4-linked glucose in the repeating unit, however, the corresponding liquid state spectrum is very different. In the liquid-state NMR spectrum of diutan two main anomeric resonances are observed, neither of which belong to diutan. These correspond to α-1,4-linked (5.37 ppm) and α-1,6-linked (4.97 ppm) glucose in amylopectin/pullulan and their assignment is supported by ^13^C HSQC data (not shown). Apparently, the diutan sample is not only polluted, but it is also a good example on an EPS which do not dissolve well, presumably because of a high molecular weight (see also alginate case above). However, from the high similarity between the ^13^C CP/MAS NMR spectra of gellan and diutan it is evident that the molecular structures are very much alike and the diutan is not polluted to a high degree.

The discrepancies between the observations from ^1^H liquid-state NMR and the ^13^C solid-state NMR reflect the low water solubility of the EPS. Only for the gellan sample a good correlation between the liquid-state and solid-state analysis was obtained. This indicates that the relatively short side chain of diutan has a major impact on the hydration properties. In the solid-state NMR spectra of gellan and diutan the integrals of the methyl resonance of rhamnose relative to the anomeric resonances (∼103 ppm) are 1:4 and 1:1.7. For gellan the ratio is just as expected, whereas the ratio is slightly lower than anticipated (1:1.33) for diutan, which supports the presence of starch/amylopectin impurities.

When comparing gellan and diutan NMR results, it seems like the presence of dimeric rhamnose side chains lowers the solubility of the EPS. Part of the reason for the lower solubility might be a higher molecular weight of diutan compared to gellan or a different packing (tertiary structure). At the demonstrated level of analysis, this is speculative, but may be further explored using a more comprehensive combined NMR and molecular modeling approach. In such a study the interaction with water could be monitored at different temperatures using both D_2_O and H_2_^17^O as sources of hydration. Due to the presence of GlcA, addition of cations may also have a great impact on the structural and function properties of EPS and the effects of cations should therefore be included in the comprehensive study as well. Previously, the impact of cations in homogalacturonans has been explored by solid-state NMR (Renard and Jarvis, 1999).

## Conclusion

Besides structure elucidation NMR also has a role in describing the functionality and in particular the hydration of the EPS. In this work, two closely related commercial EPS were characterized by preliminary multi-phase NMR spectroscopic analysis and molecular modeling with special emphasis on the hydration properties. Previously a similar approach was used to characterize the hydration behavior of native and enzymatically modified pectin (Larsen et al., 2011) which showed that the ratio of arabinan to galactan side chains were very important for the hydrophobicity of the pectin. A similar effect was observed when comparing the results for gellan and diutan. While gellan was hydrated sufficiently for analysis by ^1^H liquid-state NMR, only resonances from an amylopectin-like impurity were observed for diutan in the liquid-state NMR spectra. This illustrates that addition of the rhamnose disaccharide side chain significantly increases the hydrophobicity.

The combination of NMR and molecular modeling has great potential for providing the structural information required for relating EPS structures with rheological properties. Due to the inherent difficulties with establishing the EPS structure with the current analytical approaches we are still in need of a high throughput structure elucidation pipeline approach of novel EPS. We propose a sequential approach where NIR spectroscopy is used for identifying new EPS structures and NMR and molecular modeling for establishing the primary structure and key features of the tertiary structure of the selected new EPS. In a first approach, the EPS structure can be represented by the primary sequence and simple molecular descriptors such as persistent length and radius of gyration which can easily be derived from modeling packages such as the POLYS program. This can be achieved in semi-automated manner and when enough data becomes available EPS structures can be interfaced to databases containing related biological and physical properties. This will allow for automatic searches for correlations between structure and function through exploratory data analysis. Quantitative Structure–Activity Relationships (QSAR) or rather Quantitative Structure–Property (QSPR) relationships can then be used to explore functional changes arising from structural alterations. POLYS can thus be considered as a prototype program for opening and exploring new possibilities in the field of bacterial EPS engineering.

## Conflict of Interest Statement

The authors declare that the research was conducted in the absence of any commercial or financial relationships that could be construed as a potential conflict of interest.
